# Intensive and Efficient Design of a Two-dimensional 8 × 8 Silicon-Based Optical Phased Array Transceiver

**DOI:** 10.3390/s23239396

**Published:** 2023-11-25

**Authors:** Yaoyuan Zhang, Rui Wang, Haibo Liu, Guobin Zhao, Ming Wei, Ruitao Jiang, Kunyang Du

**Affiliations:** 1 Changchun Institute of Optics, Fine Mechanics and Physics, Chinese Academy of Sciences, Changchun 130033, China; zhangyaoyuan19@mails.ucas.ac.cn (Y.Z.); liuhaibo@ciomp.ac.cn (H.L.); weiming19@mails.ucas.ac.cn (M.W.); jiangruitao18@mails.ucas.ac.cn (R.J.); dukunyang18@mails.ucas.ac.cn (K.D.); 2 University of Chinese Academy of Sciences, Beijing 100049, China; 3 Faculty of Aviation Foundations, Aviation University of Air Force, Changchun 130022, China; zhaoguobin0129@126.com

**Keywords:** optical phased array, silicon photonic platform, transceiver integration, beam steering, photonic device design

## Abstract

In recent years, the silicon-based optical phased array has been widely used in the field of light detection and ranging (LIDAR) due to its great solid-state steering ability. At the same time, the optical phased array transceiver integration scheme provides a feasible solution for low-cost information exchange of small devices in the future. Based on this, this paper designs a two-dimensional optical phased array transceiver with high efficiency and a large field of view, which can realize a dense array with antenna spacing of 5.5 μm × 5.5 μm by using low crosstalk waveguide wiring. Additionally, it can realize the conversion between the receiving mode and the transmitting mode by using the optical switch. The simulation results show that the scanning range of 16.3° × 16.3° can be achieved in the transmitting mode, and the overall loss is lower than 10dB. In the receiving mode, we can achieve a collection efficiency of more than 27%, and the antenna array receiving loss is lower than 12.1 dB.

## 1. Introduction

The popularity of artificial intelligence promotes the rapid development of many fields such as self-driving cars [1,2], autonomous optical communication [3,4], and so on. The core of the autonomous function in these fields is to distinguish obstacles by light beam scanning [5]. However, traditional mechanical scanning technology mainly uses a group of discrete optical and electronic components [6]. The defects lie in the slow scanning speed, large volume, and high cost, so its application in many fields is limited. Therefore, the appearance of the optical phased array (OPA) provides a feasible solution for efficient beam scanning [7,8,9,10]. At the same time, the efficient compatibility of silicon on insulator (SOI) platforms and complementary metal oxide semiconductor (COMS) technology makes silicon photonic technology conducive to large-scale integration [11]. Therefore, the OPA based on a silicon photonic platform has become a research hotspot [12,13,14,15,16,17].

In recent years, most of the research on the OPA has focused on its use for signal transmission [18,19,20,21]. However, studies have shown that the use of an OPA for signal reception can receive the incident optical signal directionally through phase control. It means that it can actively receive the optical signal in the specified direction and reject the signal in other directions [22]. This function is of great value in many applications. As early as 2015, the first monolithic two-dimensional OPA transceiver was reported [23]. The optical switch was used to achieve free switching of OPA receiving and transmitting functions using the principle of optical path reversibility. The efficient integration of integrated transceiver modules confirmed the feasibility of monolithic transceivers.

At present, there are two feasible schemes to realize a two-dimensional array arrangement in silicon-based OPAs. One is to use waveguides to route directly to the antenna [24,25]. However, for traditional straight-waveguide routing due to the large crosstalk between waveguides, to achieve low crosstalk routing of 8 × 8 antenna arrays, the antenna spacing needs to be greater than 12 μm, which means that the far-field scanning range is less than 7°. The other is to use a large number of directional couplers to achieve power distribution. In 2014, a new routing scheme of OPAs was proposed achieving a scanning range of 10° × 10° with an antenna spacing of 9 μm × 9 μm [26]. In 2022, it was reported that using an external array phase shifter with an antenna spacing of 8.5 μm × 8.5 μm can achieve a scanning range of 10.5° × 10.5° [27].

All in all, for the current research work on two-dimensional planar array OPAs, it is difficult to improve the efficiency and increase the scanning range at the same time. On the one hand, the use of waveguide direct routing can greatly reduce the internal loss of the array. However, due to the crosstalk between waveguides, it is difficult to reduce the array spacing, so the scanning range of the array is severely limited. On the other hand, although the beam distribution using directional couplers can increase the scanning range to a certain extent, it will cause serious coupling loss. This defect is acceptable when only using the OPA as the transmitting module, but it is greatly amplified when using the OPA as a receiving module. In addition, the extensive use of directional couplers will increase the complexity of the internal structure of the array, and it is difficult to further reduce the array element spacing. According to current reports, for the scanning range, it is still difficult to exceed 10°, and it is difficult to achieve transmission efficiency lower than 15 dB. Therefore, we innovatively introduce the sinusoidal waveguide routing structure with low crosstalk into the OPA array, which greatly reduces the element spacing while maintaining low loss. On this basis, we propose a scheme of bidirectional incidence at both ends of the array to further reduce the element spacing.

In this paper, we propose a new type of 8 × 8 two-dimensional OPA structure that utilizes a low crosstalk waveguide routing scheme with bidirectional incidence and uses the external array phase shifter. We eliminate the extensive use of a large number of internal array beam splitters and directional couplers, which can greatly reduce the routing losses and improve energy transmission efficiency. Finally, the scanning range of 16.3° × 16.3° can be achieved with the array spacing of 5.5 μm × 5.5 μm in the transmitting mode, and the total transmission efficiency of the array is < 10 dB. In the receiving mode, the simulation shows that the receiving loss per unit area of 12.1 dB can be achieved with a collection efficiency of more than 27%. On this basis, the coherent receiving of the received optical signal can reduce stray light and other signal crosstalk as much as possible.

## 2. Simulation Principle and Calculation Method

**Figure 1:**
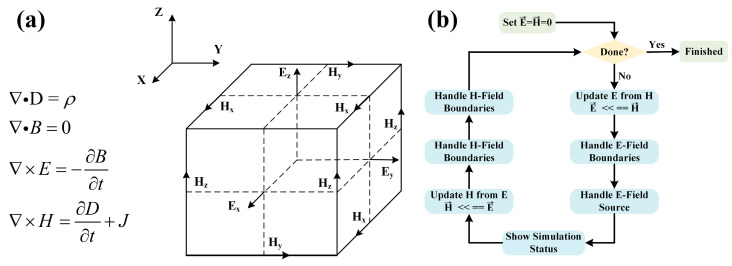
The principle of FDTD. (**a**) Discretization of Maxwell’s equations. (**b**) The specific process of FDTD.

The main working principle of the OPA is that through the cooperation of different functional device structures, one input optical signal is divided into tunable multi-beam interference in the far field, on this basis, through the reasonable control of each beam to achieve different diffraction pattern distribution in the far field. In this process, it is necessary to simulate and calculate the transmission characteristics of electromagnetic waves in all devices of the OPA. For passive devices, we mainly use the method of the finite-difference time domain (FDTD) for simulation. The main principle of this method is shown in Figure 1.

By dividing the simulation region into independent cells with certain precision, the curl equation of Maxwell equations is discretized into different equations for each cell [28], and the instantaneous electric and magnetic field components of each cell are updated through successive iterations in time, as shown in Figure 1a. Because the algorithm simulates the real-time change process of electromagnetic waves, it is directly based on Maxwell equations without introducing other approximate conditions. Therefore, it has high calculation accuracy. Figure 1b shows the specific simulation flow of the FDTD algorithm. In the simulation process, the software of Lumerical 2020 R2 is mainly used to simulate and analyze the 3D structure. The simulation time depends on the accuracy of the selected simulation grid and the size of the simulation region. For the simulation of the overall structure of the OPA with high precision, it is necessary to ensure sufficient memory of the computer.

For the simulation of active devices, we use the finite element method (FEM). It can divide the device structure into a finite number of elements and obtain the parameters of the entire structure by solving these parameter changes in each small region. The accuracy of the overall simulation depends on the precision of the region division.

## 3. Array Structure Design

**Figure 2:**
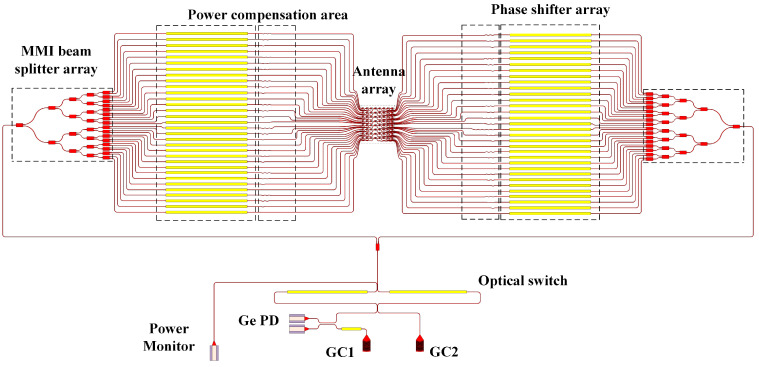
Schematic diagram of the overall structure of OPA.

The specific structure is shown in Figure 2. Two-directional wiring is used to route the optical signal, which can reduce the waveguide wiring area by half and greatly reduce the antenna spacing. The function of selecting the optical path is achieved by using a 2 × 2 Mach–Zender optical switch. It mainly uses the thermal optical effect of silicon to apply a phase difference of 0 or 2π to the two arms and further control the constructive or destructive interference on the optical path. GC2 is the incident grating, mainly responsible for transferring the 1550 nm laser signal outside the chip to the waveguide. GC1 is the reference grating that is mainly responsible for the coherent superposition of input reference light sources and received optical signals to improve detection sensitivity. In addition, to effectively determine the state of the optical switch, it is necessary to add a power monitor to obtain accurate switch information. The MMI beam splitter array mainly distributes the energy of 2 × 64 optical waveguides rationally. The power compensation area is mainly responsible for averaging the loss of each optical signal. Therefore, the energy can be evenly distributed when each waveguide is routed to the antenna. The phase shifter array can realize independent tuning of the phase of each optical signal through the thermo-optical phase shifter controlled by the TiN heater. The antenna array consists of 64 antennas uniformly arranged at a distance of 5.5 μm × 5.5 μm, and they work together to implement specific transmit or receive functions.

### 3.1. Antenna Array Design

**Figure 3:**
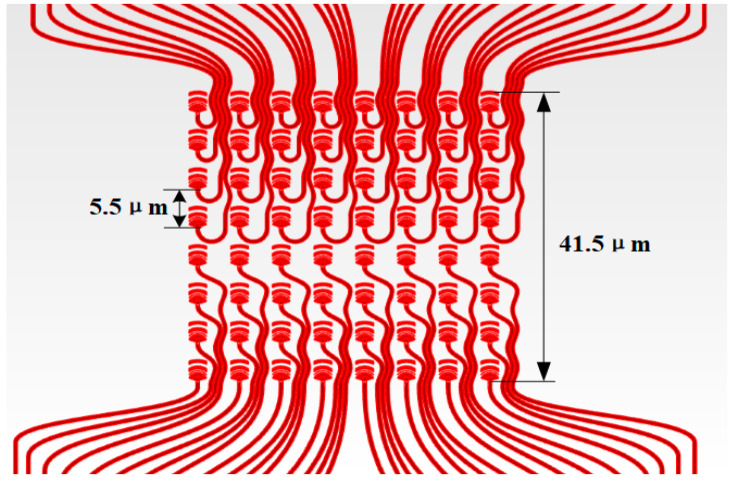
The detailed structure of the antenna array.

The specific internal routing scheme of the antenna array is shown in Figure 3. We give up the use of a large number of cross-waveguides and directional couplers to avoid greater losses and more complex waveguide wiring. Instead, separate waveguide routing is performed for each antenna unit. However, traditional straight-waveguide routing makes it difficult to achieve small waveguide spacing due to the severe crosstalk. Therefore, we adopted the design of a sinusoidal waveguide, which can minimize the crosstalk between waveguides through bending waveguide routing [29].

At the same time, we choose to route both ends of the array simultaneously to maximize space utilization. It is worth noting that because of the different lengths of the routing path to the antenna, the energy loss is also different. Therefore, it is necessary to set the power compensation area outside the array to make the energy routed to each antenna unit as evenly distributed as possible. In addition, although sinusoidal waveguide arrays have excellent anti-crosstalk capabilities, their overall loss is relatively high and is not suitable for long-distance transmission. Therefore, we still use straight waveguides for routing outside the antenna array. At the end of the array, we need to reasonably arrange a large number of S-curved waveguides to achieve a reasonable transition from small to large waveguide spacing while minimizing crosstalk.

### 3.2. Phase Shifter Array Design

The scanning of the array far-field beam is achieved by controlling the phase of each antenna to form a specific phase difference; the change in phase is mainly achieved by changing the effective refractive index of the silicon waveguide. We use the principle of the thermo-optic effect of silicon, by applying a specific voltage to the TiN heater on the upper layer of the waveguide; changing the voltage can control the temperature of the silicon waveguide and further realize the change in the effective refractive index. The thermo-optic coefficient of silicon materials is

(1) $$\frac{dn}{dT}=1.86\times 10^{-4}/K$$

The change in temperature T causes the change in refractive index n, and the relationship between phase change Δφ and effective refractive index change $\Delta n_{eff}$ is as follows:

(2) $$\Delta \varphi =\frac{2\pi }{\lambda }\Delta n_{eff}L$$

where λ is the wavelength, and L is the length of the heating waveguide.

**Figure 4:**
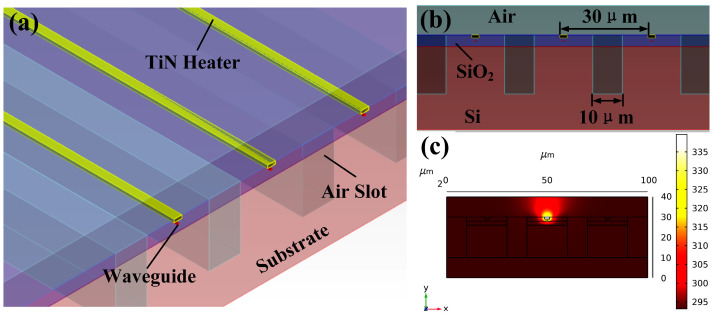
The structure of phase shifter array. (**a**) Schematic diagram of phase shifter array 3D view. (**b**) Schematic diagram of phase shifter array side view. (**c**) The result of thermal crosstalk simulation.

It is worth noting that the small heating waveguide spacing will lead to severe temperature crosstalk and inaccurate phase control. The specific phase-shifting array structure and thermal crosstalk simulation results are shown in Figure 4. We set the waveguide spacing to 30 μm and set a 10 μm-wide adiabatic air slot between the waveguides to minimize thermal crosstalk. The simulation shows that when the waveguide 2π phase change is realized, we can achieve the adjacent waveguide thermal crosstalk of >23 dB.

## 4. Functional Device Structure Design

### 4.1. Design of Low Crosstalk Waveguide

**Figure 5:**
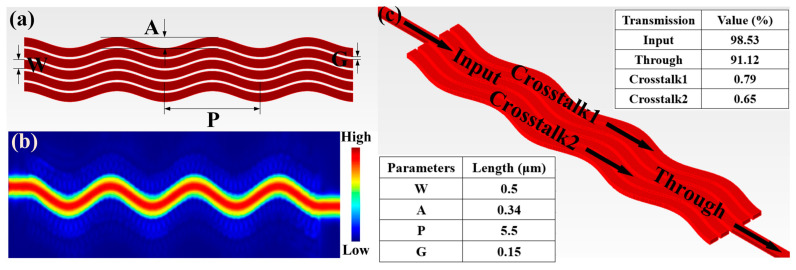
Design of low crosstalk waveguide. (**a**) Structural parameters of sinusoidal waveguide array. (**b**) Waveguide routing electric field distribution. (**c**) Waveguide routing crosstalk and insertion loss.

For traditional straight-waveguide routing arrays, the crosstalk between waveguides mainly follows the waveguide coupling theory:

(3) $$K^{2}=\frac{P_{c}}{P_{o}}={sin}^{2}\left(\frac{\pi \Delta n}{\lambda }\cdot L\right)={sin}^{2}\left(\frac{\pi }{2}\cdot \frac{L}{L_{\pi }}\right)$$

where $P_{0}$ is the input optical power, $P_{c}$ is the optical power coupled to another waveguide, L is the length of DC, Δn is the difference between the effective refractive index of the symmetric mode and the antisymmetric mode (Δ*n* = *n*_1_ − *n*_2_), and $L_{\pi }$ is the coupling length. According to Formula (1), it is difficult for the direct waveguide route to realize the low crosstalk route with small spacing and long distance. However, the crosstalk between waveguides can be reduced effectively by the reasonable bending of waveguides. Research [30] shows that the power exchange between waveguides can be effectively suppressed by setting $\Delta \beta_{bend}$ (the difference of the super mode propagation constant of the sinusoidal waveguide) to zero. $\Delta \beta_{bend}$ can be defined as

(4) $$\Delta \beta_{\mathrm{bend}}=\Delta \beta J_{0}\left(\frac{4\pi^{2}\left(W+G\right)n_{eff}A}{P\lambda_{0}}\right)$$

where Δβ is the difference of the super mode propagation constant of the straight waveguide, $J_{0}$ is the zero-order Bessel function, $n_{eff}$ is the effective refractive index of a single waveguide, W is the waveguide width, G is the waveguide spacing, A is the sinusoidal waveguide amplitude, and P is the sinusoidal waveguide period. According to this concept, we can get the approximate range of relevant parameters according to our application requirements. In the actual simulation process, we take improving transmission efficiency and reducing crosstalk between waveguides as the main optimization indexes and P, A, and G as the main optimization parameters for iterative optimization. The final optimization results are shown in Figure 5. The period P = 5.5 μm, the peak A = 0.34 μm, and the spacing G = 0.15 μm. We simulated the maximum length sinusoidal waveguide route used in our structure. The results show that sinusoidal waveguides with a total length of 19μm can achieve an insertion loss of < 0.4dB and a crosstalk of > 18.3 dB. The specific parameters are shown in Figure 5c.

### 4.2. Design of Antenna Structure

In a two-dimensional OPA, the regular arrangement of antennas is necessary to achieve the ideal far-field pattern. Therefore, the overall diffraction efficiency of the array depends on the diffraction performance of each antenna. The radiation intensity and focusing degree of each antenna will have an important impact on the far field of the array. Therefore, it is necessary to optimize antenna performance as much as possible to lay the foundation for a better far field of an OPA. At the same time, since the grating structural optical path is reversible, effective diffraction performance also represents effective receiving performance. Therefore, we only optimize the diffraction performance.

**Figure 6:**
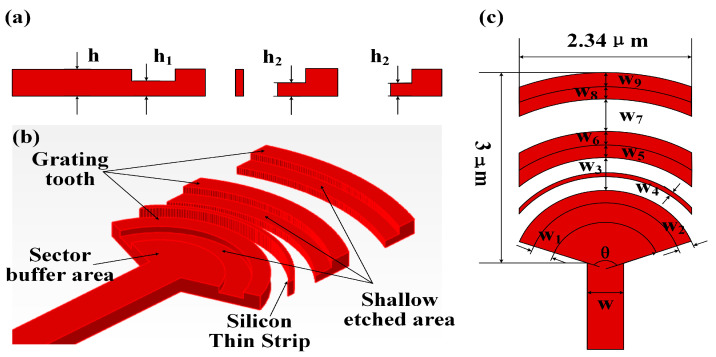
Design of antenna unit structure. (**a**) Structural parameters of the antenna side view. (**b**) Schematic diagram of antenna 3D view. (**c**) Structural parameters of antenna top view.

For the far-field focusing degree of the antenna, circular grating teeth are better than rectangular grating teeth. For the radiation intensity of the antenna, the most effective idea is to break the upper and lower symmetry of the grating. It can obtain stronger diffraction energy to free space and reduce the diffraction leakage to the substrate. In our design, we adopted our previous work and optimized the structure on this basis. The specific structure is shown in Figure 6. We added shallow etched areas in front of each grating tooth to increase the antenna’s upward diffraction efficiency. In addition, we added fan-shaped silicon thin strips in front of the grating tooth to reduce the optical back reflection energy loss. However, since the diffraction energy of the antenna is mainly concentrated in the first and second grating teeth, adding more silicon strip structures will result in losses greater than gains. Therefore, we only choose to add silicon strips before the second stage of grating teeth.

**Figure 7:**
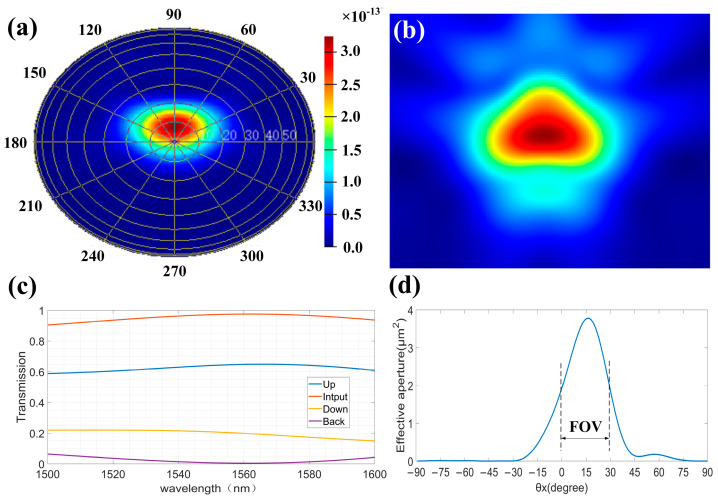
Antenna performance. (**a**) Antenna far-field distribution. (**b**) Antenna near-field distribution. (**c**) Antenna diffraction efficiency. (**d**) Antenna field of view.

On this basis, we utilize an algorithm for the iterative optimization of each design parameter. The final parameter design results are shown in Table 1. The antenna performance after optimization is shown in Figure 7. Finally, the antenna size of 2.34 μm × 3 μm can achieve more than 64% upward diffraction efficiency at 1550 nm, the loss of diffraction to the substrate is >6.8 dB, and the loss of back reflection is > 21.4 dB.

## 5. Array Performance Analysis

### 5.1. Diffraction Performance Analysis

The array radiation far field is formed by coherently superposing the far field of each antenna element. The radiation direction of the coherent beam is controlled by adjusting the phase of each element, further realizing beam deflection and scanning. The principle [31] of far-field interference is as follows.

**Figure 8:**
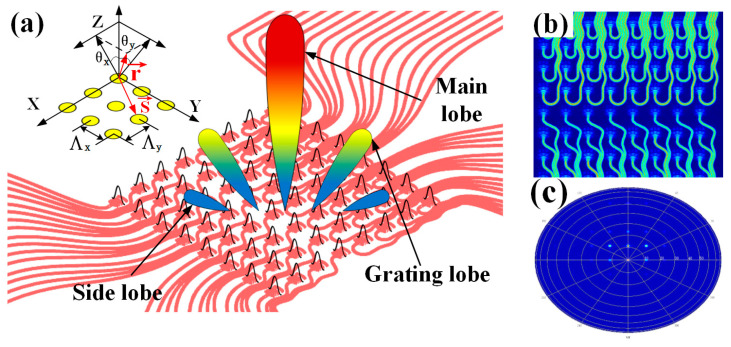
Diffraction performance of antenna array. (**a**) Schematic diagram of transmitting antenna array. (**b**) Array electric field energy distribution. (**c**) Array far-field energy distribution.

The basic structure of an $N_{x}\times N_{y}$ two-dimensional OPA is shown in Figure 8a, where $\Lambda_{x}$ and $\Lambda_{y}$ represent the spacing of antennas on the two dimensions of the OPA. $\theta_{x}$ and $\theta_{y}$ are the included angles between the propagating beam and the two direction planes, respectively. $\vec{s_{xy}}$ is the position vector of each element relative to the coordinate origin. $\vec{r}$ is the position vector of far-field radiation. According to diffraction theory,

(5) $$e(r)=\sum_{n_{x}=0}^{N_{x}-1}\sum_{n_{y}=0}^{N_{y}-1}A_{xy}\exp\left(j\beta_{xy}\right)\times F_{xy}(\theta_{x},\theta_{y})\frac{\exp\left(-jk_{0}\left|\vec{r}-\vec{s_{xy}}\right|\right)}{\left|\vec{r}-\vec{s_{xy}}\right|}$$

where $A_{xy}$ is the amplitude of each antenna, $\beta_{xy}$ is the relative phase value of the antenna, $\exp\left(j\beta_{xy}\right)$ is the phase of each element, $k_{0}$ is the free space propagation vector, and $F_{xy}(\theta_{x},\theta_{y})$ is the independent far-field distribution of each transmitting element.

In the far field, the propagation distance is much larger than the device size. The center position of the far-field distribution $F_{xy}(\theta_{x},\theta_{y})$ corresponding to each independent element is approximately the same. $\left|\vec{r}-\vec{s_{xy}}\right|$ is approximately expressed as $\left|\vec{r}-\vec{s_{xy}}\right|\approx r=R$, and the phase shift part of each element is approximately expressed as $\left|\vec{r}-\vec{s_{xy}}\right|\approx R-\vec{k}\cdot \vec{s_{xy}}$, where $\vec{k}$ is the element vector in the transmission direction. Simplify Formula (5) as

(6) $$e(r)=F(\theta_{x},\theta_{y})\frac{\exp\left(-jk_{0}R\right)}{R}\times \sum_{n_{x}=0}^{N_{x}-1}\sum_{n_{y}=0}^{N_{y}-1}A_{xy}\exp\left(j\beta_{xy}\right)\exp\left(-j\vec{k}\vec{s_{xy}}\right)$$

Formula (6) is divided into two parts. $F\left(\theta_{x},\theta_{y}\right)\frac{\exp\left(-jk_{0}R\right)}{R}$ is the diffraction distribution of each independent element in the far field, which depends on the characteristics of each element, called the element factor. $\sum_{n_{x}=0}^{N_{x}-1}\sum_{n_{y}=0}^{N_{y}-1}A_{xy}\exp\left(j\beta_{xy}\right)\exp\left(-j\vec{k}\vec{s_{xy}}\right)$ is the interaction between arrays, reflecting a series of array parameters, called array factors. Therefore, the matrix factor $T\left(\theta_{x},\theta_{y}\right)$ is written as

(7) $$T\left(\theta_{x},\theta_{y}\right)=\sum_{n_{x}=0}^{N_{x}-1}\sum_{n_{y}=0}^{N_{y}-1}A_{xy}\times \exp\left(j\beta_{xy}\right)\exp\left(-j\vec{k}\vec{s_{xy}}\right)$$

When the amplitude strength and phase of each element in the OPA are identical, let $A_{xy}=A$ and $\beta_{xy}=\beta$. Because the array spacing on each dimension is the same, $S_{xy}=n_{x}\cdot \Lambda_{x}\cdot u_{x}+n_{y}\cdot \Lambda_{y}\cdot u_{y}$, and the array factor can be written as

(8) $$\begin{array}{l} T\left(\theta_{x},\theta_{y}\right)=A\exp\left(j\beta \right)\exp\left(-j\gamma \right)\\ \times \frac{\sin\left[\frac{N_{x}}{2}k_{0}\Lambda_{x}\sin\theta_{x}\right]}{\sin\left[\frac{1}{2}k_{0}\Lambda_{x}\sin\theta_{x}\right]}\frac{\sin\left[\frac{N_{y}}{2}k_{0}\Lambda_{y}\sin\theta_{y}\right]}{\sin\left[\frac{1}{2}k_{0}\Lambda_{y}\sin\theta_{y}\right]} \end{array}$$

where *γ* can be expressed as

(9) $$\begin{array}{l} \gamma =\left(N_{x}-1\right)k_{0}\Lambda_{x}\sin\theta_{x}/2\\ +\left(N_{y}-1\right)k_{0}\Lambda_{y}\sin\theta_{y}/2 \end{array}$$

It can be seen from the formula that the X direction and the Y direction exist independently. The following only takes the X direction as an example to analyze. For Formula (8), the maximum value will be obtained only when the numerator and denominator terms are close to zero; therefore,

(10) $$\frac{1}{2}k_{0}\Lambda_{x}\sin\theta_{x}=q\pi$$

(11) $$\sin\theta_{x}=q\cdot \frac{\lambda }{\Lambda_{x}}$$

where q is an integer, representing the number of interference peaks. When q=0, the peak corresponding to the interference maximum is called the main lobe, the interference peak corresponding to the other q values is called the high-order gate lobe, and the weak signal between the main lobe and the gate lobe is called the side lobe.

In the OPA, to realize beam scanning, it is necessary to introduce phase difference into the array to deflect the beam and introduce phase difference $\beta_{xy}=n_{x}\cdot \Delta \varphi_{x}+n_{y}\cdot \Delta \varphi_{y}$ into Formula (7), so that

(12) $$\begin{array}{l} T\left(\theta_{x},\theta_{y}\right)=A\exp\left(j\gamma \right)\\ \frac{\sin\left[\frac{N_{x}}{2}\left(k_{0}\Lambda_{x}\sin\theta_{x}-\Delta \varphi_{x}\right)\right]}{\sin\left[\frac{1}{2}\left(k_{0}\Lambda_{x}\sin\theta_{x}-\Delta \varphi_{x}\right)\right]}\frac{\sin\left[\frac{N_{y}}{2}\left(k_{0}\Lambda_{y}\sin\theta_{y}-\Delta \varphi_{y}\right)\right]}{\sin\left[\frac{1}{2}\left(k_{0}\Lambda_{y}\sin\theta_{y}-\Delta \varphi_{y}\right)\right]} \end{array}$$

Similarly, the phase term can be expressed as

(13) $$\gamma =\frac{N_{x}-1}{2}\left(k_{0}\Lambda_{x}\sin\theta_{x}-\Delta \varphi_{x}\right)+\frac{N_{y}-1}{2}\left(k_{0}\Lambda_{y}\sin\theta_{y}-\Delta \varphi_{y}\right)$$

Take the X direction as an example, when $T\left(\theta_{x},\theta_{y}\right)$ takes the maximum value:

(14) $$\frac{1}{2}\left(k_{0}\Lambda_{x}\sin\theta_{x}-\Delta \varphi_{x}\right)=q\pi$$

where q is an integer, representing the different levels of diffraction in the far field, and when $\Lambda_{x}>\lambda /2$, the far-field spot scanning range can be obtained:

(15) $$\theta_{x}\approx \arcsin\left(\frac{\lambda }{\Lambda_{x}}\right)$$

The expression in the Y direction is similar to the above equation. When each antenna element is the same, the scanning angle only depends on the antenna element spacing. According to the calculation, we can realize the far-field scanning range of 16.3° × 16.3°.

**Figure 9:**
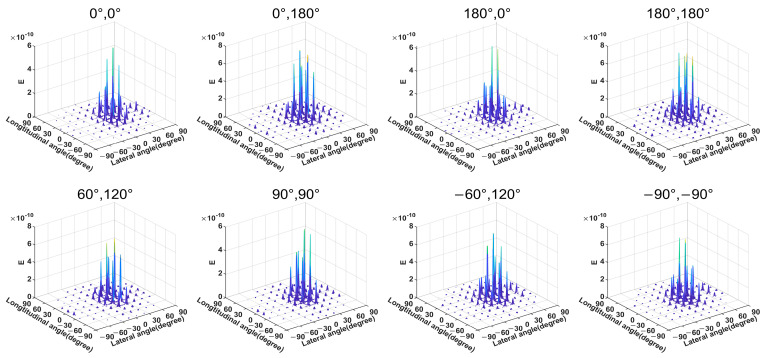
Far-field 3D scanning diagram of the array under different phase differences.

On this basis, we conduct a simulation of our antenna array, and Figure 8b shows the array’s electric field distribution. Figure 8c shows the array far-field distribution when no phase difference is applied. In addition, we perform a simulation analysis of the far-field deflection when applying different phase differences, as shown in Figure 9.

In addition, due to reducing the complexity of the internal wiring of the array, the energy transmission efficiency of the array was greatly improved. The simulation shows that in the transmitting mode, without considering the thermal loss of the phase shifter, the overall optical loss is less than 10.1 dB, including the waveguide transmission loss of 2 dB/cm and a series of curved waveguide losses. There is no special design for other devices in our simulation. The loss of a single MMI is 0.41 dB, the loss of the grating coupler is 1.93dB, and the optical loss of the optical switch is 0.86 dB. According to the research, the maximum power of silicon material cannot exceed 100 mW under the premise of maintaining functional stability. Therefore, the maximum output power in our structure will not exceed 10 mW. The design is safe and suitable in its application field. In some applications with high-power requirements, SiN materials can be considered as an alternative.

### 5.2. Reception Performance Analysis

For the receiving module, using an antenna array for receiving can realize the directional reception of the incident beam compared with traditional large-scale grating coupler reception. The principle is to achieve the constructive or destructive interference of optical signals entering the array by adjusting the phase of each path. It means that it can actively choose to receive optical signals in the specified direction while rejecting signals in other directions.

However, due to the weak receiving signal, the most important thing for the receiving module is to achieve high energy utilization as much as possible, and our structure mainly improves this indicator from the following three aspects.

The first is to improve the antenna conversion efficiency, and we optimized the antenna structure above, which can finally achieve an efficiency of more than 64%. At the same time, for the overall antenna array, there will be a large number of optical signals entering the substrate directly in the receiving mode. Therefore, it is necessary to reduce the spacing of the antenna to achieve the most compact array, which is consistent with the transmitting module’s goal of increasing the scanning range. We calculated that under the antenna spacing of 5.5 μm, we can achieve an effective receiving area of more than 27%.

Secondly, it is necessary to reduce the loss of optical signals after entering the array, and the use of a large number of directional couplers and cross-waveguides will inevitably cause serious energy loss. Therefore, we use the sinusoidal waveguide routing directly. According to the simulation, we can reduce the loss by more than 6 dB compared with the cross-waveguide structure.

Finally, limited by the threshold value of silicon material power, when the laser signal reaches the detector after a series of losses, the signal intensity will become very weak. Therefore, coherent detection of the signal must be carried out by inputting reference light to further improve reception sensitivity and achieve efficient reception of the signal.

**Figure 10:**
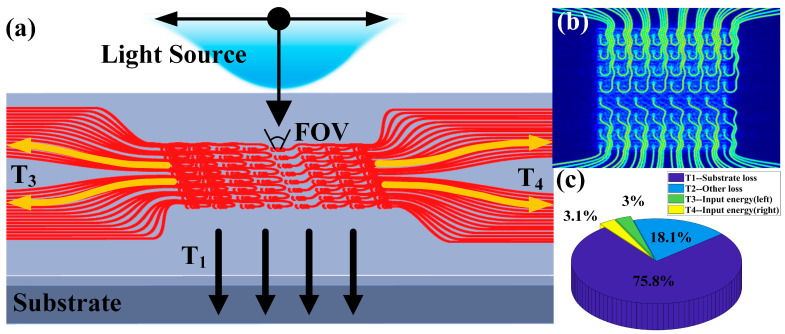
Antenna array receiving performance. (**a**) Schematic diagram of receiving antenna array. (**b**) Array electric field energy distribution. (**c**) Receive array transmission efficiency.

On this basis, we carried out a simulation analysis for signal reception. We simulated the external light source incident antenna array as shown in Figure 10. We tested the conversion efficiency of the 8 × 8 antenna array when the light source was vertically incident on the optical signal. The results show that only considering the internal components of the antenna array, there is a power loss of 1.2 dB, the radiation of which enters the substrate directly. The conversion efficiency of the input optical signal on both sides of the antenna array is 15.2 dB and 15 dB, respectively, as shown in Figure 10c.

**Figure 11:**
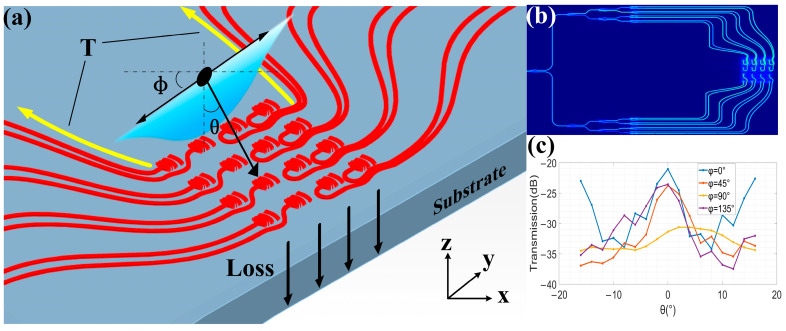
The 4 × 4 antenna array receiving performance. (**a**) Schematic diagram of receiving 4 × 4 antenna array. (**b**) Array electric field energy distribution. (**c**) The transmission efficiency of the receiving array at different incident angles.

In addition, we simulated a 4 × 4 simplified model of the antenna array to verify the active signal selection function of the OPA, as shown in Figure 11. Pitch angle θ is the angle between the x and z planes, and the azimuth angle φ is the angle between the y and z planes. When the light source is vertically incident without adding phase difference, the electric field distribution inside the receiving array is shown in Figure 11b. We can see that the antenna array has no directional selection feature for the beam, and the beam selection function is mainly through destructive or constructive interference when the signal beam is combined.

Then, we set the external light sources to be incident at different angles, and the array terminal receiving power is shown in Figure 11c. The data show that under the condition of fixed phase difference, the receiving efficiency of the array for signals in the specified direction is significantly better than in other directions, which confirms the active selection function of the phased array for beams.

## 6. Manufacturing and Scalability

**Figure 12:**
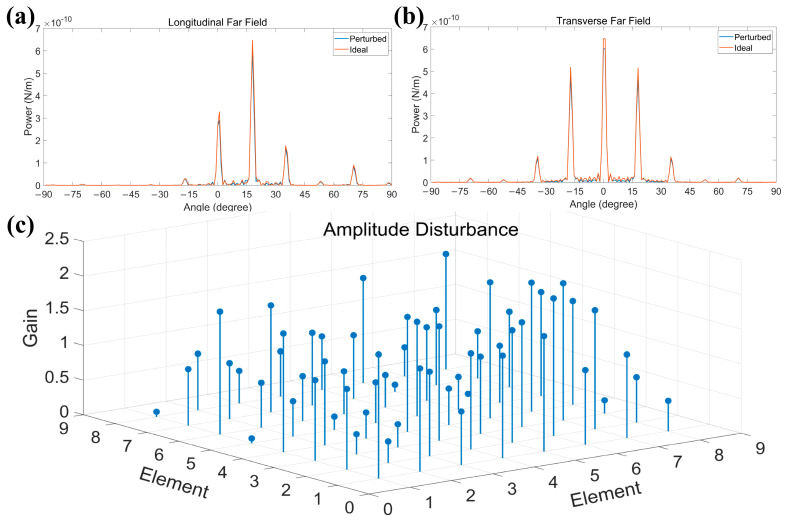
Array amplitude tolerance analysis. (**a**) Longitudinal far-field comparison of the array before and after disturbance. (**b**) Transverse far-field comparison of the array before and after disturbance. (**c**) Random amplitude disturbance in the range of 0 to 2.

Our design scheme involves a large number of micro-nanostructures of the SOI platform. It requires high-precision semiconductor growth and etching process. At present, with the rapid development of intelligent industry, the relevant manufacturing process is retaining gradually improving accuracy. At present, the conventional manufacturing accuracy is about 130 nm. However, the highest precision process has been able to reach the process node of 28 nm. Therefore, the manufacturing process can fully meet our design requirements. We used sinusoidal waveguides with 150 nm pitch in our structure, and etching heights as low as 108 nm were used in the antenna design. Therefore, we need to use 90 nm or higher precision etching processes in the remanufacturing process. In addition, a series of manufacturing errors are inevitably introduced during the manufacturing process, such as changes in the thickness and surface roughness of the waveguide. This change will inevitably lead to an increase in internal structure crosstalk and loss and eventually affect the amplitude and phase changes of each antenna. As a result, it is necessary to use thermal oxidation and wet corrosion technology, which can effectively improve the roughness of the waveguide surface. For the phase change of each antenna element caused by manufacturing, we can carry out phase calibration during the experiment to compensate for the possible phase deviation. However, it is difficult to compensate for the introduced antenna amplitude changes. Therefore, we introduce random amplitude perturbations to simulate the possible influence of process errors on the design results, as shown in Figure 12.

By analyzing the far-field distribution of the array before and after interference, it is concluded that when the manufacturing error affects the amplitude of the antenna, the main lobe energy of the far field will be dispersed. However, through simulation analysis, the overall array has a good ability to resist amplitude interference, and it will not have too much influence on the beam control ability of the array.

Due to the limitation of our computer computing power, we only carried out simulation analysis for an 8 × 8 array, and the analysis showed that excellent performance could be obtained. With the increase in the number of array elements, the array spacing will inevitably increase. According to the calculation for the 16 × 16 array, the antenna spacing of 8.1 μm can be achieved, and the scanning range of more than 10° can still be achieved while maintaining high transmission efficiency. However, for larger arrays, there are still limitations in the scanning range, which is similar to the traditional direct waveguide routing array.

All in all, our design scheme provides a new research idea for the design of the OPA two-dimensional array structure, which has obvious advantages over direct waveguide routing arrays. It has excellent performance in small and medium-sized arrays and has good application prospects in some scenarios with low requirements for array scale, such as information exchange of handheld smart devices, intelligent robots, virtual reality (VR) technology, and other fields. At the same time, due to the limited power threshold of Si material, it is difficult to apply in some high-power scenarios. Therefore, we can consider applying our design scheme to SiN or other silicon composites for relevant scenarios. In addition, because Si is a short-band-gap semiconductor, it is difficult to achieve high luminous efficiency. To achieve all-silicon integration of the OPA, further research on silicon light sources is necessary.

## 7. Conclusions

In conclusion, we designed an ultra-compact two-dimensional silicon-based OPA transceiver, which enables arbitrary switching of OPA transmission and reception functions through optical switches. We used an external array phase shifter and 64-line waveguide routing in the array, eliminating the use of the independent phase shifter and a large number of directional couplers inside the antenna array, greatly improving the power transmission efficiency. The simulation results show that the wide field of view scanning of 16.3° × 16.3° can be achieved with the compact antenna element spacing of 5.5 μm × 5.5 μm in transmitting mode, and the overall loss does not exceed 10dB. In the receiving mode, we verified the directional selection function of OPA receiving for the beam and realized the loss of <12.1 dB from free space to the waveguide with a collection efficiency of more than 27%.

## Figures and Tables

**Table 1:** Antenna parameter data.

θ	h	h_1_	h_2_	w	w_1_	w_2_	w_3_	w_4_	w_5_	w_6_	w_7_	w_8_	w_9_
140°	0.22 μm	0.123 μm	0.108 μm	0.5 μm	0.3 μm	0.208 μm	0.5 μm	0.057 μm	0.2 μm	0.222 μm	0.37 μm	0.15 μm	0.2 μm

## Data Availability

Data are contained within the article.
